# Investigating the Role of Race and Stressful Life Events on the Smoking Patterns of Pregnant and Postpartum Women in the United States: A Multistate Pregnancy Risk Assessment Monitoring System Phase 8 (2016–2018) Analysis

**DOI:** 10.1007/s10995-023-03773-7

**Published:** 2023-09-22

**Authors:** Rauta Aver Yakubu, Kobi V. Ajayi, Shubhecchha Dhaurali, Keri Carvalho, Anna Kheyfets, Blessing Chidiuto Lawrence, Ndidiamaka Amutah-Onukagha

**Affiliations:** 1 Maternal Outcomes for Translational Health Equity Research (M.O.T.H.E.R) Lab, Center for Black Maternal Health and Reproductive Justice, Tufts University School of Medicine, Boston, MA 02111 USA; 2 College for Public Health and Social Justice, Saint Louis University, St. Louis, MO 63104 USA; 3 Texas A&M University, College Station, TX 77845 USA; 4 School of Arts & Sciences, Tufts University, Medford, MA 02155 USA; 5 School of Medicine, Tufts University, Boston, MA 02111 USA

**Keywords:** Smoking, Perinatal, Psychosocial, Stress, Disparities, PRAMS, Maternal morbidities

## Abstract

**Objective:**

To examine the smoking patterns of women who experienced stressful life events and the impact of racial disparities on the relationship between stressful life events, and prenatal/ postpartum smoking.

**Methods:**

The study analyzed data from the Pregnancy Risk Assessment Monitoring System Phase 8 (2016–2018) survey across five states (CT, LA, MA, MO, WI). Four stressful life event categories were created using thirteen affiliated questions: financial, trauma, partner, and emotional. We assessed: 1) the association between smoking and stressful life events, 2) the impact of race on the relation between smoking and stressful life events, and 3) the long-term effects of smoking on health by assessing the association between smoking and maternal morbidity. Bivariate statistics and multivariate Poisson regression models were conducted.

**Results:**

A total of 24,209 women from five states were included. 8.9% of respondents reported smoking during pregnancy, and 12.7% reported smoking postpartum. There was a significant association between all stressful life events and smoking. Trauma stressful life event had the strongest association with smoking during pregnancy (adjusted PR=2.01; CI: 1.79-2.27) and postpartum (adjusted PR= 1.80; CI: 1.64-1.98). Race and stressful life event interaction effects on smoking had varied significant findings, but at least one racial/ ethnic minority group (Black, Hispanic, Asian) had a higher smoking prevalence than non-Hispanic White per stressful life event category. Lastly, the prevalence of maternal morbidity was higher for smoking during pregnancy (adjusted PR= 1.28; CI: 1.19-1.38) and postpartum (adjusted PR= 1.30; CI: 1.22-1.38) compared to no smoking.

**Conclusions for Practice:**

Culturally congruent, multi-disciplinary care teams are needed to address both clinical and social needs to reduce stressful life events and smoking. Screenings for stress should be standardized with a referral system in place to provide ongoing support.

## Significance

*What is already known?* Smoking during the perinatal period is associated with adverse maternal and infant health outcomes. Psychosocial stress is associated with the prevalence of smoking during the perinatal period. Racial minorities have a higher prevalence of psychosocial stress due to longstanding systemic factors including racism.

*What this study adds?* Most studies assessing stressful life events and smoking focus on single states or preconception and prenatal periods. This novel study is a multi-state analysis assessing both the prenatal and postpartum periods. Furthermore, this study provides an overview of the types of stress different racial groups are affected by and recommendations for effective smoking cessation, stress screening, and stress reduction methods.

## Introduction

Cigarette smoking (hereinafter referred to as only “smoking”) is one of the most preventable causes of perinatal mortality and morbidity (Tong et al., 2013; Yang et al., 2014). A recent national study in the United States found an overall decrease in smoking prevalence during pregnancy from 9.2% to 2010 to 6.9% in 2017 (Azagba et al., 2020). Similar patterns have been reported in earlier studies in the United States and Canada, showing a steady decline in smoking during pregnancy (Gilbert et al., 2014; Hansen et al., 2018). Despite the decrease in maternal smoking, evidence suggests that the rates of smoking during pregnancy have remained steady or continued to increase among minority and systematically marginalized subpopulations (Hansen et al., 2018; Scheffers-van Schayck et al., 2019). Azagba and colleagues found higher smoking prevalence among American Indian/Alaskan Natives, women ages 20–24, and those with high school diplomas (2020). Smoking during pregnancy can also extend into the postpartum period. Researchers found that approximately half of women who smoke before pregnancy continue to smoke during pregnancy (Kia et al., 2018). Of the 20% of women who quit smoking during pregnancy, 50% relapsed within six months of childbirth (Kia et al., 2018). Although people smoke for a myriad of reasons, there is evidence that increased stressful life events are associated with unhealthy habits and risky behaviors, such as smoking, which serve as coping mechanisms (March of Dimes, 2015; van Dijk et al., 2021).

Stressful life events are experiences associated with psychosocial stressors such as financial stressors, trauma (e.g., intimate partner violence), systemic racial and/or gender discrimination, chronic stress, or day-to-day hassles (Allen et al., 2019; Beutel et al., 2018). A former study using the Pregnancy Risk Assessment Monitoring System (PRAMS) surveillance data found that survivors of intimate partner violence were significantly more likely to smoke before and throughout pregnancy than those who have not experienced this form of stressful life event (Alhusen et al., 2018). This study and others show that smokers who experienced trauma were more likely to smoke and relapse than smokers who did not experience trauma (Allen et al., 2019; Ogneva-Himmelberger & Haynes, 2020). In addition to smoking, chronic stress weakens the immune system and can lead to illnesses, such as uterine infection, hypertension, and heart disease and may increase the risk for maternal morbidity (Bekhbat & Neigh, 2018; Low et al., 2010; March of Dimes, 2021; Wadhwa et al., 2011).

Abundant research has documented racial and ethnic disparities and the prevalence of psychosocial stressors (Carvalho et al., 2022; Giurgescu & Misra, 2018; Lu & Chen, 2004). Black and American Indian/Alaska Native women reported the highest number of stressful life events 12 months before delivery (Lu & Chen, 2004). Pregnant Black women were more likely to report experiences of racial discrimination and bias, psychological stress, living in disadvantaged neighborhoods, experiencing symptoms of depression, using avoidance coping mechanisms, and having lower levels of social support compared to white women (Giurgescu & Misra, 2018). The evidence indicates that smoking and stressful life events are persistent among women in racial and ethnic minority groups, contributing to the increased maternal morbidity and mortality rate among these populations.

Considering the significant impact of smoking and stressful life events on maternal health outcomes, researchers need to continue investigating the mechanisms that enable these occurrences. The objectives of this study were to examine the smoking patterns of women who experienced stressful life events, the impact of racial disparities on the relationship between stressful life events and smoking in the prenatal and postpartum periods, and how smoking affects the prevalence of maternal morbidity.

## Methods

### Data Source and Participants

This study analyzed Phase 8 (2016–2018) data from the PRAMS survey. PRAMS is a population-based surveillance system funded by the Centers for Disease Control and Prevention in partnership with state health departments. PRAMS collects data on maternal behaviors before, during, and after live birth pregnancies. The PRAMS survey includes core questions administered across all participating states and state-specific questions (Centers for Disease Control and Prevention, 2021). PRAMS data across five states (Connecticut, Louisiana, Massachusetts, Missouri, and Wisconsin) were included. These states were selected because their state surveys included questions on the stressful life events assessed in this study.

## Measures

### Outcome Variables

Two questions from the PRAMS core questions were used for the outcome measures:

- “In the last three months of your pregnancy, how many cigarettes did you smoke on an average day?” (for during pregnancy/ prenatal).
- “How many cigarettes do you smoke on an average day now?” (for postpartum period).

Both questions originally contained seven response options from 41 cigarettes or more to “I didn’t smoke then” or “I don’t smoke now” but were recoded to two binary outcome variables for analysis (Alhusen et al., 2018; Allen et al., 2019). For the third objective, which was to estimate the effect of smoking on maternal morbidity, one binary (no/yes) outcome variable was created for women who indicated “yes” to having gestational diabetes, gestational hypertension, and/or prenatal depression. Postpartum depression questions were also included in maternal morbidity. Questions related to postpartum depression included:

- “I have felt down, depressed. or hopeless.”
- “I have had little interest or pleasure in doing things I usually enjoyed.”

### Key Independent Variable: Stressful Life Events

Psychosocial stress was assessed using the stressful life events questions asked in PRAMS. Mothers responded to 14 yes/no questions about their experiences with specific stressful life events during the 12 months before their infant’s birth. The life events were:

1. A close family member was very sick and had to go into the hospital.
2. I got separated or divorced from my husband or partner.
3. I moved to a new address.
4. I was homeless or had to sleep outside, in a car, or a shelter.
5. My husband or partner lost their job.
6. I lost my job even though I wanted to go on working.
7. My husband, partner, or I had a cut in work hours or pay.
8. I was apart from my husband or partner due to military deployment or extended work-related travel.
9. I argued with my husband or partner more than usual.
10. My husband or partner said they didn’t want me to be pregnant.
11. I had problems paying the rent, mortgage, or other bills.
12. My husband, partner, or I went to jail.
13. Someone very close to me had a problem with drinking or drugs.
14. Someone very close to me died.

We excluded the third question asking whether mothers had moved to a new address because moving to a new address might be positive or negative. As such, only 13 of the 14 stressful life event questions were analyzed for this study. Based on previous studies, we created binary yes/no variables for each of the 13 questions and grouped each question into one of four categories: partner-related stress (2, 8, 9, 10); trauma-related stress (4, 12, 13); financial-related stress (5, 6, 7, 11); and emotional-related stress (1, 14) (Ahluwalia et al., 2001; Allen et al., 2019; Stone et al., 2015; Lawrence et al., 2022). In addition to stressful life events, we also developed a multilevel variable for the total number of stressful events based on the 13 stressful life events we used for analysis: no stress, 1 to 3 stressors, 4 to 6 stressors, and more than 7 stressors to assess the potential gravity of multiple stressful life events on health outcomes (Stone et al., 2015; Lawrence et al., 2022).

### Covariates

**Fig. 1:**
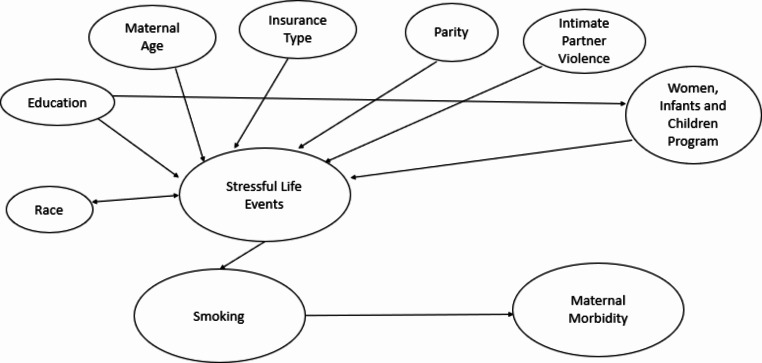
Directed acyclic graph of causal effects of stressful life events on perinatal smoking and maternal morbidity

Covariates were selected based on the directed acyclic graphs theory attributed to the causal effects of stressful life events on smoking as well as the causal effect of smoking on maternal morbidity (Fig. 1). Selected covariates included maternal age, race and ethnicity, mother’s education, parity (i.e., number of total pregnancies that reached 20 weeks gestation or more), pregnancy insurance, participation in the Special Supplemental Nutrition Program for Women, Infants, and Children (WIC), and intimate partner violence. Maternal age was coded as younger than 20 years, 20 to 24 years, 25 to 34 years, and 35 years and older. Race and ethnicity were grouped as non-Hispanic Black, non-Hispanic white, Hispanic, non-Hispanic American Indian/Alaskan Native, non-Hispanic Asian, and non-Hispanic Others (race unknown). Education was categorized as less than 12 years, 12 years, and greater than 12 years. Marital status was dichotomized as married versus unmarried. Parity was defined as zero and one or more. Insurance coverage during recent pregnancy was categorized as private insurance, Medicaid, and no insurance. Since the variables related to insurance type varied across states in PRAMS, we selected insurance options that were consistent across the states included in this study. WIC was assessed as a binary yes or no variable. Finally, intimate partner violence in the 12 months before and during pregnancy was measured by a binary yes or no variable.

### Statistical Analysis

The analysis included mothers from the five states in this study. Respondents from these states were included if they answered the relevant questions related to smoking in the last three months of pregnancy (during pregnancy/ prenatal) and/or after pregnancy (postpartum period). Chi-square tests at p < 0.05 were used to assess descriptive statistics and meaningful differences. All covariates of interest were retained to align with the causal effect directed acrylic graph model. Poisson regression was used to determine independent associations between each of the stressful life events categories and smoking. Another regression using the four-level variable for number of stressors was completed with “no stress” being the reference level. Prevalence ratios (PR) at a 95% confidence interval (CI) was determined based on Poisson regression output. All analyses were completed using STATA v. 17.0, accounting for the complex survey design of PRAMS and included appropriate survey weights for population-based estimates.

## Results

### Study Characteristics

In the study sample (N = 24,209), 8.9% of respondents reported smoking during pregnancy, and 12.7% reported smoking in the postpartum period (Table 1). The majority identified as white at 64.2%, 17.1% Black, and 12.9% Hispanic. Over half (64.8%) of respondents had over 12 years of education, 60% were 25 to 34 years of age or married, and 52.5% used public insurance (Medicaid) while pregnant. About 60% had reported at least one stressful life event, 27.8% experienced a partner-related life event, 14.5% experienced a trauma-related life event, 31.2% experienced an emotional-related life event, and 31% experienced a financial-related life event.

**Table 1:** Characteristics of participants, stratified by smoking during pregnancy and postpartum

		Study characteristics stratified by smoking during the last three months of pregnancy			Study characteristics stratified by smoking now (N = Count Value)		
Variable	Sample N (Weighted %)	No Smoking N = 21,990 (91.1%)**	Yes Smoking N = 1,841 (8.9%)**	P value*	No Smoking N = 21,142 (87.3%)**	Yes Smoking N = 2,678 (12.7%)**	P value*
Partner-related life event				< 0.001			< 0.001
No	16,998 (72.2)	15,970 (74.3)	914 (50.7)		15,484 (74.9)	1,397 (53.7)	
Yes	6.834 (27.8)	5,877 (25.7)	905 (49.3)		5,522 (25.1)	1,255 (46.3)	
Trauma-related life event				< 0.001			< 0.001
No	20,534 (85.5)	19,323 (88.1)	1,066 (59.3)		18,719 (88.6)	1,664 (64.2)	
Yes	3,285 (14.5)	2,512 (11.9)	752 (40.7)		2,275 (11.4)	987 (35.8)	
Emotional-related life event				< 0.001			< 0.001
No	16,586 (68.8)	15,495 (70.1)	976 (55.5)		15,002 (70.7)	1,469 (56)	
Yes	7,247 (31.2)	6,352 (29.9)	844 (44.5)		6,005 (29.3)	1,183 (44)	
Financial-related life event				< 0.001			< 0.001
No	16,119 (69)	15,177 (71.3)	831 (45.3)		14,722 (71.9)	1,287 (49.2)	
Yes	7,700 (31)	6,659 (28.7)	987 (54.7)		6,272 (28.1)	1,365 (50.8)	
Number of stressors				< 0.001			< 0.001
No stress	9,534 (40.2)	9,163 (42.4)	301 (17.2)		9,163 (42.4)	301 (17.2)	
1–3 stressors	11,076 (46.4)	10,155 (46.5)	853 (45.9)		10,155 (46.5)	853 (45.9)	
4–6 stressors	2,687 (11.2)	2,182 (9.6)	479 (27.2)		2,182 (9.6)	479 (27.2)	
More than 7 stressors	556 (2.28	367 (1.6)	187 (9.7)		367 (1.6)	187 (9.7)	
Race/Ethnicity				< 0.001			< 0.001
Non-Hispanic Black	6,906 (17.1)	6,203 (17.4)	562 (14.3)		5,890 (17.1)	872 (16.7)	
Non-Hispanic White	9,427 (64.2)	8,301 (63.1)	1,016 (76.2)		7,935 (63.1)	1,377 (72.5)	
Hispanic	4,480 (12.9)	4,255 (13.6)	153 (5.2)		4,127 (13.8)	278 (6.7)	
Non-Hispanic AI/AN	299 (1.07)	236 (0.9)	58 (2.7)		219 (0.8)	75 (2.5)	
Non-Hispanic Asian	1,998 (3.9)	1,933 (4.2)	29 (1.1)		1,913 (4.3)	49 (1.2)	
Non-Hispanic Other	327 (0.80)	304 (0.8)	15 (0.5)		304 (0.9)	14 (0.4)	
Education				< 0.001			< 0.001
less than 12 years	2,953 (11.1)	2,423 (9.8)	464 (23.5)		2,270 (9.4)	614 (21.7)	
12 years	5,682 (24.1)	4,845 (22.3)	720 (41.7)		4,514 (21.5)	1,052 (41.8)	
over 12	15,374 (64.8)	14,533 (67.9)	652 (34.8)		14,179 (69.1)	997 (36.4)	
Maternal age				< 0.001			< 0.001
Younger than 20	1,077 (4.03)	968 (4.0)	78 (3.8)		911 (3.8)	135 (5.1)	
20–24	4,257 (18.1)	3,756 (17.4)	415 (24.5)		3,508 (16.9)	661 (26.0)	
25–34	14,270 (60)	12,979 (60.1)	1,096 (59.7)		12,515 (60.4)	1,551 (57.3)	
35 or over	4,605 (17.9)	4,287 (18.5)	252 (11.9)		4,208 (18.8)	331 (11.5)	
Marital status				< 0.001			< 0.001
Not married	10,667 (40)	9,025 (36.4)	1,431 (75.6)		8,418 (35.2)	2,034 (72.2)	
Married	13,530 (60)	12,956 (63.6)	407 (24.4)		12,715 (64.8)	641 (27.8)	
Insurance while pregnant				< 0.001			< 0.001
Private	10,232 (51.2)	9,899 (43)	220 (14.8)		9,735 (56.3)	384 (18.1)	
Medicaid	11,769 (46.8)	10.091 (54.9)	1,466 (83.9)		9.470 (41.7)	2,078 (80.7)	
None	416 (1.9)	380 (2.04)	27 (1.3)		375 (2.1)	32 (1.2)	
WIC				< 0.001			< 0.001
No	13.666 (64.4)	12,870 (67)	607 (37.7)		12,595 (68.2)	883 (38.9)	
Yes	10,149 (35.6)	8,769 (33)	1,199 (62.3)		8,218 (31.8)	1,738 (61.1)	
Parity				< 0.001			< 0.001
0	9,388 (38.7)	8,793 (40.1)	456 (24.6)		8,484 (40.2)	760 (28.7)	
1 or more	14,780 (61.3)	13,163 (59.9)	1,379 (75.4)		12,624 (59.8)	1,912 (71.3)	
IPV				< 0.001			< 0.001
No	23,331 (98.1)	21,479 (98.6)	1,662 (92.7)		20,666 (98.7)	2,468 (94.4)	
Yes	484 (1.87)	337 (1.4)	141 (7.3)		312 (1.3)	165 (5.6)	
State				< 0.001			< 0.001
CT	5,401 (22.3)	5,098 (12.0)	224 (6.6)		4,949 (12.2)	372 (7.0)	
LA	3,713 (15.3)	3,356 (20.3)	324 (21.6)		3,154 (19.8)	527 (24.5)	
MA	5,867 (24.2)	5,599 (24.4)	194 (12.3)		5,477 (24.7)	316 (13.6)	
MO	4,709 (19.5)	4,008 (22.5)	643 (35.3)		3,792 (22.1)	854 (34.1)	
WI	4,519 (18.7)	3,929 (20.8)	456 (24.1)		3,770 (21.1)	609 (20.9)	
Maternal morbidity				< 0.001			< 0.001
No	14,962 (64)	14,026 (65.8)	799 (45.6)		13,599 (66.4)	1,224 (47.6)	
Yes	9,076 (36)	7,959 (34.2)	1,042 (54.4)		7,538 (33.6)	1,454 (52.4)	

* Significance is < 0.05; **Percentages are rounded and may not equal 100

AI/AN: American Indian/ Alaska Native

CT: Connecticut

IPV: Intimate partner violence

LA: Louisiana

MA: Massachusetts

MO: Missouri

N: count value

WI: Wisconsin

WIC: Special Supplemental Nutrition Program for Women, Infants, and Children

Most respondents who smoked during pregnancy reported a financial-related life event (54.7%). 49% had at least one partner-related life event, 40.7% had a trauma-related life event, and 44.5% had an emotional-related trauma event. The majority were unmarried at 75.6%, and 41.7% had 12 years of education. Of those who smoked, 83.9% utilized Medicaid and 62.3% used the WIC program. Of the states assessed, Missouri had the highest number of people who smoked both during pregnancy (35.3%) and postpartum period (34.1%). Among those who smoked in the postpartum period, half reported a financial-related life event, the highest frequency among stressful life events.

### Association Between Stressful Life Events and Smoking

After adjusting for race, education, maternal age, insurance during pregnancy, WIC, parity, and intimate partner violence, a significant and positive association still existed between stressful life events and smoking during pregnancy and the postpartum period (Table 2). Trauma-related life events had the strongest association of all life event categories during pregnancy (adjusted PR = 2.01; CI: 1.79–2.27) and the postpartum period (adjusted PR = 1.80; CI: 1.64–1.98) followed by financial-related stress during pregnancy and emotional-related stress during the postpartum period.

**Table 2:** Association between stressful life events and smoking

	Model 1: During Pregnancy				Model 2: Postpartum			
	Unadjusted		Adjusted*		Unadjusted		Adjusted*	
Stressful Life Event	PR (95% CI)	P value**	PR (95% CI)	P value**	PR (95% CI)	P value**	PR (95% CI)	P value**
Partner-related life event	2.53 (2.25–2.83)	< 0.001	1.44 (1.28–1.62)	< 0.001	2.24 (2.04–2.45)	< 0.001	1.36 (1.24–1.50)	< 0.001
Trauma-related life event	4.06 (3.63–4.54)	< 0.001	2.01 (1.79–2.27)	< 0.001	3.30 (3.01–3.61)	< 0.001	1.80 (1.64–1.98)	< 0.001
Emotional-related life event	1.77 (1.58–1.99)	< 0.001	1.44 (1.29–1.61)	< 0.001	1.73 (1.56–1.90)	< 0.001	1.44 (1.32–1.58)	< 0.001
Financial-related life event	2.69 (2.39–3.01)	< 0.001	1.48 (1.31–1.67)	< 0.001	2.30 (2.10–2.52)	< 0.001	1.34 (1.22–1.48)	< 0.001
Number of Stressors								
No stress	Reference		Reference		Reference		Reference	
1–3 stressors	2.30 (1.95–2.72)	< 0.001	1.66 (1.40–1.96)	< 0.001	2.15 (1.89–2.45)	< 0.001	1.62 (1.42–1.84)	< 0.001
4–6 stressors	5.70 (4.78–6.80)	< 0.001	2.46 (2.04–2.96)	< 0.001	4.76 (4.15–5.47)	< 0.001	2.24 (1.94–2.60)	< 0.001
More than 7 stressors	9.94 (8.09–12.20)	< 0.001	3.31 (2.64–4.15)	< 0.001	7.04 (5.93–8.37)	< 0.001	2.68 (2.22–3.23)	< 0.001

* Adjusted for education, maternal age, insurance during pregnancy, WIC, parity, intimate partner violence

** Significance is < 0.05

CI: confidence interval

PR: prevalence ratio

The more stressful events a woman experienced, the higher the prevalence of smoking during the pregnancy and postpartum period. During pregnancy, for more than seven stressors, smoking prevalence reduced from PR = 9.94 (CI: 8.09–12.20) to PR = 3.31 (CI: 2.64–4.15) after adjusting for covariates (education, maternal age, insurance during pregnancy, WIC, parity, intimate partner violence). In the postpartum period, smoking prevalence reduced from PR = 7.04 (CI: 5.93–8.37) to adjusted PR = 2.68 (CI: 2.22–3.23). Overall, a minimal difference exists in adjusted smoking prevalence when assessing the number of stressful events experienced between the prenatal and postpartum period.

### Association Between Race, Stressful Life Events, and Smoking

Interactions between stressful life events and race were assessed with white as the reference for smoking patterns during the pregnancy and postpartum period (Table 3).

**Table 3:** Association between race, stressful life events, and smoking

	Model 1: During Pregnancy		Model 2: Postpartum	
	Adjusted*		Adjusted*	
**Interactions**	PR (95% CI)	P value**	PR (95% CI)	P value**
**Partner-related life event**				
Non-Hispanic White	Reference		Reference	
Non-Hispanic Black	1.13 (0.86–1.48)	0.380	1.13 (0.91–1.39)	0.271
Hispanic	**1.91 (1.28–2.86)**	**0.002**	**1.65 (1.23–2.23)**	**< 0.001**
Non-Hispanic AI/AN	0.77 (0.44–1.36)	0.367	0.85 (0.42–1.66)	0.489
Non-Hispanic Asian	0.52 (0.20–1.39)	0.194	0.84 (0.42–1.66)	0.616
Non-Hispanic Other	1.49 (0.38–5.78)	0.567	1.23 (0.27–5.50)	0.788
**Trauma-related life event**				
Non-Hispanic White	Reference		Reference	
Non-Hispanic Black	**1.52 (1.16–1.98)**	**0.002**	1.23 (1.00-1.53)	0.052
Hispanic	**2.04 (1.36–3.06)**	**0.001**	**1.76 (1.29–2.39)**	**< 0.001**
Non-Hispanic AI/AN	1.28 (0.69–2.37)	0.429	2.84 (1.51–5.34)	0.185
Non-Hispanic Asian	**2.65 (1.14–6.12)**	**0.023**	**2.84 (1.51–5.34)**	**< 0.001**
Non-Hispanic Other	2.71 (0.66–11.05)	0.164	1.74 (0.36–8.34)	0.487
**Emotional-related life event**				
Non-Hispanic White	Reference		Reference	
Non-Hispanic Black	**1.35 (1.04–1.76)**	**0.023**	**1.29 (1.05–1.58)**	**0.014**
Hispanic	1.21 (0.79–1.85)	0.374	1.33 (0.98–1.81)	0.070
Non-Hispanic AI/AN	1.05 (0.60–1.83)	0.865	0.95 (0.59–1.51)	0.818
Non-Hispanic Asian	0.93 (0.40–2.18)	0.869	1.04 (0.54–2.03)	0.901
Non-Hispanic Other	**8.78 (2.08–36.93)**	**0.003**	**8.15 (2.03–32.76)**	**0.003**
**Financial-related life event**				
Non-Hispanic White	Reference		Reference	
Non-Hispanic Black	0.93 (0.71–1.22)	0.617	0.96 (0.78–1.18)	0.697
Hispanic	0.97 (0.65–1.45)	0.617	1.13 (0.84–1.52)	0.434
Non-Hispanic AI/AN	0.91 (0.52–1.61)	0.755	0.94 (0.59–1.51)	0.796
Non-Hispanic Asian	1.21 (0.51–2.85)	0.663	1.26 (0.67–2.38)	0.470
Non-Hispanic Other	2.32 (0.60–8.98)	0.223	**5.40 (1.27–22.98)**	**0.022**

* Adjusted for education, maternal age, insurance during pregnancy, WIC, parity, intimate partner violence

** Significance is < 0.05 (significant findings are in bold)

AI/AN = American Indian/Alaska Native

CI: confidence interval

PR: prevalence ratio

For emotional life stressors, only those identified as non-Hispanic Black and Other had a significant association during pregnancy (Black: adjusted PR = 1.35; CI: 0.22–0.32; Other: adjusted PR = 8.78; CI: 2.08–36.93) and postpartum period (Black: adjusted PR = 1.29; CI: 1.05–1.58; Other: adjusted PR = 8.15; CI: 2.03–32.76). For trauma stressors during pregnancy, Blacks, Hispanics, and Asians had a significant, positive association with smoking, and Asians had the highest smoking prevalence (adjusted PR = 2.65; CI: 1.14–6.12). In the postpartum period, Hispanics, Asians, and Others had significant findings of traumatic stressful life events and higher smoking prevalence than white women. For partner-related stressors, only Hispanics had a significant association with smoking during pregnancy (PR = 1.91; CI: 1.28–2.86) as well as the postpartum period (adjusted PR = 1.65; CI: 1.22–2.23).

### Association Between Smoking and Maternal Morbidity

As seen in Table 4, participants who smoked during pregnancy were more likely to experience maternal morbidity than those who did not smoke during pregnancy (adjusted PR = 1.28; CI: 1.19–1.38). Likewise, women who smoked during the postpartum period were more likely to report maternal morbidity than those who did not smoke (adjusted PR = 1.30; CI: 1.22–1.38). These outcomes were based on adjusting for race, education, maternal age, insurance during pregnancy, WIC, parity, and intimate partner violence.

**Table 4:** Association between smoking and maternal morbidity

	Model 1: Maternal morbidity during pregnancy		Model 2: Maternal morbidity postpartum	
	Adjusted*		Adjusted*	
Variable	PR (95% CI)	P value**	PR (95% CI)	P value**
*Smoking*				
No	Reference		Reference	
Yes	1.28 (1.19–1.38)	< 0.001	1.30 (1.22–1.38)	< 0.001

* Adjusted for race, education, maternal age, insurance during pregnancy, WIC, parity, intimate partner violence

** Significance is < 0.05

CI: confidence interval

PR: prevalence ratio

## Discussion

This study examined the impact of major stressful life events on smoking during the pregnancy and postpartum period, racial disparities in smoking prevalence, and the effect of smoking on maternal morbidity. Key results aligned with the study hypotheses and showed (1) a significant, positive association between stressful life events and smoking during the pregnancy/postpartum period; (2) for stressful life events and race, at least one racial and ethnic minority group had a higher prevalence of smoking compared to white women; and (3) when assessing the association between smoking and maternal morbidity, we found that maternal morbidity prevalence was higher for those who smoked both during the pregnancy and postpartum period compared to those who did not smoke.

Previous PRAMS studies on stressful life events align with our findings on smoking prevalence. Allen et al. (2019) assessed smoking and stress from preconception to the postpartum period and found that half of women quit smoking during pregnancy, but 44% relapsed in the postpartum period. In addition, when individuals experienced stressful life events, their odds of smoking during pregnancy increased (Allen et al., 2019). Similar findings are present in related studies from the last 12 years (Carmichael & Ahluwalia, 2000; Salm Ward et al., 2017; Stone et al., 2015; Tong et al., 2013), and our study adds to the literature by providing more recent data trends. This longstanding relationship between smoking and stress alludes to potential correlations between socioeconomic status and smoking as a coping mechanism. Most respondents who smoked during or after pregnancy in our study and in the literature utilized Medicaid (during pregnancy), a commonly used proxy for socioeconomic status (Ogneva-Himmelberger & Haynes, 2020; Wiggert et al., 2016).

Our analysis of stressful life events, race, and smoking adds to the PRAMS literature on racial disparities and coping with racial discrimination. Substance-use-related disorders are often referenced to as a “self-help” or coping strategy for stress related to chronic exposure to systemic racism (Borrell et al., 2013; Farahmand et al., 2020; Giurgescu et al., 2020; Vu et al., 2019). Racial minorities are exposed to higher stressful life events compared with white women, increasing the likelihood that they will use smoking as a coping mechanism (Drake et al., 2018; Kondracki, 2019). Thus, smoking patterns from the perspective of stressful life events show that racial minorities have a higher smoking prevalence compared to white women (Drake et al., 2018; Kondracki, 2019; Slopen et al., 2012). No studies that we are aware of have assessed the interaction of stressful life events and race on smoking, but studies have assessed different forms of self-perceived stress. In a study assessing stress, chronic disease, and health behaviors among Hispanic communities, perceived stress was associated with increased odds of smoking (Gallo et al., 2014). Our study is also based on perceived stress; respondents are noting their perceptions of stressful events from the PRAMS questionnaire. Furthermore, in a study on the California Behavioral Risk Factor Surveillance System, women who experienced racial and/or ethnic discrimination were at greater odds of smoking (Plascak et al., 2018). However, Plascak et al. assessed all women, including those who did not recently deliver, while our study focused on women who had a recent live birth only.

Although most of our findings aligned with the literature, our findings on Asian stressful life events did not. A prior stressful life events-related PRAMS study showed that Asians had the highest household income and a smaller number of stressful events than other racial groups (Liu et al., 2016). This contrasts with our findings on financial stressful life events and race: Asians had an increased prevalence of smoking during the postpartum period. Although overall demographic trends in the United States show Asians as having the least financial stressors, Asians now have the largest economic divide within their racial group compared to others: Mongolians have the highest poverty rate among Asian groups in the United States (25%), while Indians have the lowest (6%) (Budiman & Ruiz, 2021; Kochhar & Cilluffo, 2018). Future studies should assess the determinants causing stressful life events among different racial groups and how they affect the prevalence of coping behaviors.

Finally, our analysis of the association between smoking and maternal morbidity explains the long-term impact of smoking and stressful life events on maternal morbidity. Experiencing stressful life events increase the prevalence of smoking, which may lead to maternal morbidity. Our findings support this hypothesis: the prevalence of maternal morbidity was higher when a woman smoked during pregnancy and the postpartum period. Increased risk of chronic diseases such as stroke, myocardial infarction, and pulmonary embolism are listed in the literature (Lee & DeFranco, 2020; Roelands et al., 2009). Although pregnant women who smoked in those studies were more likely to have experienced many different forms of morbidity (e.g., asthma, pneumonia, bronchitis), they were less likely to have experienced gestational diabetes or pre-eclampsia (Roelands et al., 2009). This contrasts with our analysis that focused on gestational diabetes and hypertension for maternal morbidity. As a result, our findings call for more research to understand the distinct pathways between stressful life events and smoking on maternal morbidity. Also, strategies to address the factors associated with smoking and stressful life events and reduction interventions for women may hold promise.

### Strengths and Limitations of Our Study

Our study provides meaningful contributions to psychosocial stress, substance use disorder, and pregnancy-related literature. Most studies previously published in this area are now five to 10 years old. Our findings provide more recent evidence of the need for perinatal smoking and stressful life events interventions. Furthermore, most stressful life events-related studies focus on one state; this study covers multiple states that have high and low maternal morbidity rates. In addition to providing a multistate analysis, most pregnancy studies focus on the preconception and prenatal period while ours includes the “fourth trimester”: the postpartum period.

Despite these strengths, our study has some limitations. Because our study only analyzed five states (Connecticut, Louisiana, Massachusetts, Missouri, Wisconsin), the results may not be generalizable to the entire country. We recommend that the next iteration of the PRAMS survey integrate a standardized stress scale into the core questionnaire so data will be available for all states. Women whose pregnancy did not result in a live birth (i.e., pregnant women whose births resulted in spontaneous abortions, ectopic pregnancies, or stillbirths) were omitted from the analysis. In addition, the results of this cross-sectional study are subject to recall and misclassification bias because participants typically complete the PRAMS survey within the range of 2 to 6 months postpartum. Finally, the stressful life events categories used in the analysis are based on prior studies but do not encompass the full range of potential stressful life events women may experience.

## Conclusions for Practice

As seen from the findings in this study, stressful life events have major implications for women’s health during the perinatal period. More research is needed on potential root causes for racial differences in stressful life events and smoking to develop culturally appropriate treatment models. Preventive measures to reduce stressful life events and smoking include implementing multidisciplinary, trauma-informed, team-based care models. These models of care help address the multifaceted root causes of stressful life events and smoking. One way to implement these models of care effectively is to integrate peer support staff (community health workers, doulas, peer recovery coaches) into the medical care team. Peer support staff may be inclined to provide trauma-informed care because they are more likely to have had shared lived experiences with the people they serve. This can pave the way to achieving effective culturally congruent care. Additionally, multidisciplinary models help connect people at risk of stressful life events to social determinants of health-related resources to mitigate potential stressors. To ensure effective team-based care models are sustained, peer support staff need to take ongoing professional development on trauma informed and culturally congruent care. When women adhere to recommendations, they may be more inclined to reduce smoking, reduce stressful life events, and build a trusting relationship with their care team.

Furthermore, standardized stress screenings need to be a part of perinatal care visits. Currently, the American College of Obstetricians and Gynecologists recommends “a full assessment of mood and emotional well-being” but does not state that providers should screen for their patients’ level of stress (American College of Obstetricians and Gynecologists, 2018). Entities that screen for stressful life events must also have an efficient referral system to provide culturally congruent support services. Future studies should assess the effectiveness of stressful life event supports on smoking trends and maternal morbidity over time. Moreover, future studies should evaluate differences among specific states used in analysis and compare findings to confounding factors that may be affecting the state differences.

## Data Availability

Data is available upon request and approval from CDC PRAMS

## Code availability

Available upon request
